# Prevention and Treatment of Chemotherapy-Induced Alopecia: What Is Available and What Is Coming?

**DOI:** 10.3390/curroncol30040275

**Published:** 2023-03-25

**Authors:** Tongyu C. Wikramanayake, Nicole I. Haberland, Aysun Akhundlu, Andrea Laboy Nieves, Mariya Miteva

**Affiliations:** Dr. Phillip Frost Department of Dermatology & Cutaneous Surgery, University of Miami Miller School of Medicine, Miami, FL 33136, USA

**Keywords:** scalp cooling, minoxidil, photobiomodulation therapy, bimatoprost

## Abstract

Millions of new cancer patients receive chemotherapy each year. In addition to killing cancer cells, chemotherapy is likely to damage rapidly proliferating healthy cells, including the hair follicle keratinocytes. Chemotherapy causes substantial thinning or loss of hair, termed chemotherapy-induced alopecia (CIA), in approximately 65% of patients. CIA is often ranked as one of the most distressing adverse effects of chemotherapy, but interventional options have been limited. To date, only scalp cooling has been cleared by the US Food and Drug Administration (FDA) to prevent CIA. However, several factors, including the high costs not always covered by insurance, preclude its broader use. Here we review the current options for CIA prevention and treatment and discuss new approaches being tested. CIA interventions include scalp cooling systems (both non-portable and portable) and topical agents to prevent hair loss, versus topical and oral minoxidil, photobiomodulation therapy (PBMT), and platelet-rich plasma (PRP) injections, among others, to stimulate hair regrowth after hair loss. Evidence-based studies are needed to develop and validate methods to prevent hair loss and/or accelerate hair regrowth in cancer patients receiving chemotherapy, which could significantly improve cancer patients’ quality of life and may help improve compliance and consequently the outcome of cancer treatment.

## 1. Introduction

Globally, 18.1 million people were diagnosed with cancer (excluding non-melanoma skin cancers) in 2020, and this number is projected to rise to 26 million in 2040 [1,2]. By 2040, the number of patients requiring first-course chemotherapy each year will increase by approximately 50% to 15.0 million [2]. Among patients receiving chemotherapy, an estimated 65% are expected to develop chemotherapy-induced alopecia (CIA) and possible changes in hair pigmentation, texture, quantity, and growth [3,4]. While many common targeted therapies and some immunotherapies are classified as chemotherapy in the National Cancer Database (NCDB), in this review we focus on CIA induced by cytotoxic drugs [5].

CIA is one of the most visible and dreaded adverse effects of systemic cytotoxic drugs for both male and female patients [4,6,7]. Cytotoxic drugs kill rapidly proliferating cells, not only the cancer cells but also highly proliferative healthy cells such as hematopoietic cells, intestinal epithelial cells, and hair follicle (HF) keratinocytes during the growth phase called anagen. Within the anagen HF, the main targets of cytotoxic drugs are the highly proliferative keratinocytes in the hair matrix at the bottom of the HF and their pigmentary system, leading to rapid apoptosis and hair shaft breakage/shedding [8]. Because up to 90% of scalp HFs are in the growth phase at any given time, scalp hair loss is often massive within weeks of the initiation of chemotherapy [9]. The risk of CIA and the degree of hair loss differ substantially based on the medication, dose, frequency, duration, and route of administration. The incidence of CIA is more than 80% for antimicrotubule agents (e.g., paclitaxel), 60–100% for topoisomerase inhibitors (e.g., doxorubicin), more than 60% for alkylators (e.g., cyclophosphamide), and 10–50% for antimetabolites (e.g., 5-fluorouracil and leucovorin) [10]. Combination therapy can further increase the incidence. Beard, eyebrow, and eyelashes can also be affected, and the severity of these hair defects may vary by the specific drugs and treatment regimens [4,7]. While alopecia is generally reversible 3–6 months after cessation of chemotherapy (the new hair may show different texture and color), some patients experience no hair regrowth or only partial hair regrowth. Permanent CIA (pCIA) is diagnosed when there is no hair regrowth or incomplete hair regrowth 6 months after chemotherapy cessation. The incidence of pCIA is dose- and schedule-related and has been associated with docetaxel given at doses of 75 mg/m^2^ or higher per cycle, and less commonly paclitaxel [11,12,13,14]. In a study of 383 patients treated for breast cancer in the UK, pCIA was reported by 23.3% of patients receiving docetaxel (among a total of 245 patients) and 10.1% paclitaxel (among a total of 138 patients) (*p* < 0.01) [13]. In a study of 61 patients treated for breast cancer in Korea, the incidences of pCIA at 6 months and 3 years were 39.5% and 42.3%, respectively [12]. As a result, pCIA (together with vision damage) is at the center of thousands of lawsuits against docetaxel (sold under Taxotere) manufacturer Sanofi-Aventis. Depletion of HF keratinocyte stem cells via apoptosis, DNA damage, and epithelial-mesenchymal transition were detected upon chemotherapy in human scalp HF organ culture and may be one of the underlying mechanisms of pCIA [15,16].

Given that scalp and facial hair are key elements of good health, beauty, and youth in social communication [17], CIA has significant negative impact on patients’ self-esteem, body image, sexuality, and overall quality of life [4,7,8,18]. In a study of 179 male and female patients who developed CIA, 101 (56.4%) patients felt that alopecia was the worst side effect of chemotherapy, 129 (72%) patients said hair loss was affecting their social life, and 37 (20.6%) patients used hair accessories to hide the hair loss [6]. Cancer patients would even consider rejecting life-saving chemotherapy for fear of CIA [4,19]. Being a public sign of the illness, the visibility of alopecia makes it difficult for patients to keep their cancer status private; the moment alopecia becomes apparent is often the moment of public recognition of the patient’s cancer treatment [20]. Consequently, patients with CIA may begin to perceive certain changes in the attitudes towards them, ranging from sympathy to rejection [20]. There are excellent recent reviews on the clinical features, diagnosis, pathobiology of CIA, and CIA effects on quality of life [3,4,9,21,22,23,24].

Despite the severe negative impact of CIA on a large number of patients, preventative and treatment options have been limited for CIA. To date, only one approach, i.e., scalp cooling, has been cleared by the US Food and Drug Administration (FDA) in 2015 to prevent CIA. In recent years, topical and oral minoxidil, photobiomodulation therapy (PBMT), platelet-rich plasma (PRP) injections, and other oral and topical treatments that have shown efficacy in promoting hair growth in other alopecia disorders are explored to stimulate hair regrowth after CIA. Here we review the current state of CIA prevention and treatment and discuss new approaches that are being tested in clinical trials. Given the negative psychological and emotional impact of CIA, approaches that successfully prevent hair loss or accelerate hair regrowth will significantly improve patients’ quality of life and may help improve compliance and consequently the outcome of cancer treatment.

## 2. CIA Prevention: Scalp Cooling and Topical Vasoconstrictors

### 2.1. Scalp Cooling Systems and Cold Caps

Scalp cooling has been used since the late 1970s to prevent CIA, and the scalp cooling system DigniCap was the first device cleared by the U.S. Food and Drug Administration (FDA) for the prevention of CIA in 2015 in breast cancer patients [9]. Scalp cooling prevents or reduces CIA via two main mechanisms: first, by reducing the scalp temperature using cold air, gel packs, or electronically cooled caps, local vasoconstriction reduces drug inflow to the scalp, consequently limiting the local concentration of chemotherapeutic agents in the HFs; second, the low temperature also helps to reduce cellular metabolism in the HFs, making them less vulnerable to the antimitotic and antimetabolic effects of chemotherapeutic drugs [20]. Additionally, low temperature-induced cell cycle arrest at the G0/G1 phase, increased HSP70 accumulation to protect cells from stresses, and reduced cell apoptosis have also been shown as potential mechanisms of HF protection against CIA [25].

Currently, there are several FDA-cleared scalp cooling systems available to patients, as well as cold caps that are not yet cleared by the FDA (Table 1). The cooling systems are mobile cooling units that pump circulating cold coolant to cool the scalp, with the temperature accurately controlled by a computer. They are operated by healthcare providers (Table 1). The cold caps use 3–8 caps containing coolant gels that can be re-frozen quickly. Temperature is carefully monitored, and the caps are changed manually. They are self-administered by the patient’s companion (Table 1). While some costs may be reimbursed by some insurance plans for the FDA-cleared cooling systems, others are not, and costs and access remain an important issue for many patients.

To achieve effective protection, the scalp must attain a subcutaneous (1–2 mm) temperature below 22 °C, which is equivalent to an epicutaneous temperature of 19 °C, although greater preventive effects could be achieved with temperatures close to 15 °C [20,26]. Scalp cooling begins approximately 30 min before chemotherapy starts, continues during the infusion, and must continue for a set period after the conclusion of treatment. The post-infusion cooling time depends on the pharmacokinetics of the chemotherapeutic agents and doses used, typically 60–180 min. The cooling cap remains on the scalp for another 5–10 min to return to room temperature. Adverse effects such as a feeling of coldness, neck and shoulder discomfort, headache, scalp pain, forehead pain, nausea, and/or light-headedness and dizziness have been reported, but are temporary [27]. However, patient compliance is high, and efficacy of protection from hair loss by scalp cooling has been increasingly demonstrated over the past 20 years, with average success rates of 50–70% [27]. It is important to note that efficacy for hair preservation depends on chemotherapy type, e.g., higher efficacy is associated with a taxane-alone regimen, and much lower efficacy is associated with an anthracycline-based regimen [28]. Over 70% of current clinical trials aimed at prevention and treatment of CIA are testing scalp cooling devices, alone or in combination with other approaches (Table 2).

While the FDA has cleared the expanded use of scalp cooling systems to patients with solid tumors undergoing chemotherapeutic protocols associated with a high risk of developing CIA, scalp cooling is not applicable to all chemotherapy regimens. For example, patients receiving platinum derivatives who develop severe peripheral neuropathies, which limit their tolerance to cold, should avoid scalp cooling [29]. Patients with hematological tumors should also avoid scalp cooling for their increased risk of scalp metastasis [9]. Furthermore, scalp cooling devices should be avoided for patients with cold agglutinin disease, cryoglobulinemia, and posttraumatic cold injury due to the risk of triggering local or generalized attack [30].

### 2.2. Topical Calcitriol

Vitamin D, partly synthesized within the keratinocytes in the presence of ultraviolet-B radiation and partly obtained from diet, plays an important role in dermatology and dermatotherapeutics due to its anti-inflammatory and immunomodulatory properties, as well as regulation of keratinocyte differentiation and proliferation [31]. Vitamin D is also believed to stimulate hair growth and anagen initiation, and its deficiency has been closely linked to various types of alopecia, including telogen effluvium, androgenetic alopecia, alopecia areata, trichotillomania, and scarring alopecia [31,32]. Although previous studies have failed to detect positive effects of calcitriol on patients with CIA—in fact, application of calcitriol (1,25-dihydroxyvitamin D3) caused a pruritic irritative dermatitis in five of the eight patients—further investigation of the drug was suggested because patients did not experience any systemic toxic effects [33]. Topical calcitriol was applied to 23 female breast and gynecologic cancer patients who were receiving a taxane-based chemotherapy, and a reduction of alopecia of >50% was observed in 8 patients at Week 7 (NCT01588522) (Table 2) [34]. The safe dosage was determined as 80 μg/mL [34].

### 2.3. Keratinocyte Growth Factor (KGF)

Keratinocyte growth factor (KGF), also called fibroblast growth factor 7 (FGF7), is a potent mitogen that regulates the migration and differentiation of various epithelial cells and protects them from various insults under stress conditions [35]. Intradermal injections of a bioengineered hair formulation containing various growth factors, including KGF, into the scalp, once every 3 weeks for a total of eight sessions, resulted in significant reduction in hair fall, decreased number of vellus hairs, and increased number of terminal hairs and shaft diameter [36]. In addition, in a neonatal rat model of cytosine arabinoside-induced alopecia, pretreatment with recombinant KGF alone induced a dose-dependent cytoprotective effect, abrogating as much as 50% of the alopecia [37]. Furthermore, in human scalp HF organ culture, KGF pretreatment slightly, but significantly, inhibited HF apoptosis and dystrophy induced by 4-hydroperoxycyclophosphamide (4-HC), a key cyclophosphamide metabolite [38]. A study to investigate KGF hair serum for the prevention of CIA (NCT04554732) was recently completed (Table 2).

### 2.4. Topical Vasoconstrictors

Topical vasoconstrictors have also been shown to be effective in protecting against CIA and oral mucositis, but only in animal models to date. In one study, when epinephrine was topically applied to the dorsal skin of neonatal rats, a 95% coat retention (suppression of alopecia) was observed in rats treated with N-nitroso-N-methylurea, and a 16% coat retention was observed in rats treated with systemic Cytoxan [39]. The promising result justifies further testing to determine whether vasoconstrictors can protect against CIA in humans. A major advantage of vasoconstrictors over scalp cooling is that they can be applied when necessary, while scalp cooling is only administered at the time of infusion. Because the half-life of chemotherapeutic drugs is longer than their infusion time, vasoconstriction through scalp cooling at the time of infusion cannot prevent their toxic effects in the subsequent days/weeks. Additionally, topical vasoconstrictors can be used for patients who are not good candidates for scalp cooling [9].

### 2.5. Low Intensity Ultrasound (LIUS)

Recently, low intensity ultrasound (LIUS) was shown to reverse the cytotoxic damage of paclitaxel on microtubules in cultured cells [40,41]. Given their excellent safety profile and availability as inexpensive home devices, it is important to explore the potential of LIUS application to prevent CIA.

## 3. CIA Treatment

It often takes several months for hair to grow back after CIA. Several agents, physical methods, and injections that have shown efficacy in promoting hair regrowth in other types of alopecia—such as alopecia areata and androgenetic alopecia—have been explored for their ability to expedite hair regrowth after CIA. These include topical and oral minoxidil, bimatoprost/prostaglandin analog, photobiomodulation therapy (PBMT), platelet-rich plasma (PRP), spironolactone, and other agents, as well as complementary and alternative treatments. These agents, physical methods, and injections are discussed below.

### 3.1. Minoxidil

Minoxidil is a pyrimidine N-oxide that acts as an antihypertensive vasodilator [42]. It induces angiogenesis by upregulating vascular endothelial growth factor expression and activating prostaglandin endoperoxide synthase 1, which can stimulate hair growth [43]. Moreover, minoxidil alters the temporal aspects of the hair cycle by extending the HF growth phase—termed anagen—while shortening the relative quiescent phase—termed telogen [9,44]. Minoxidil was approved by the FDA to treat male and female pattern hair loss in 1988, and 2% or 5% topical minoxidil solution or 5% foam has been widely used to promote hair regrowth, including off-label use for other types of alopecia, including CIA [9,44,45,46]. In a double-blind randomized controlled study, women who were administered topical minoxidil 2% twice a day 4 months post-chemotherapy showed a statistically significant decrease (*p* = 0.03) for the alopecic period, reducing it from 137 days for the placebo to 87 days [21,47]. Additionally, a recent controlled study found topical minoxidil to be effective in promoting hair regrowth after taxane-based chemotherapy, even in the case of pCIA [11]. Furthermore, oral minoxidil has also shown efficacy. A low dosage of oral minoxidil, starting at 1.25 mg daily, has also been used in the treatment of pCIA [48]. Oral minoxidil treatment would be particularly useful for patients with poor compliance to topical minoxidil due to scalp irritation or hair texture. A review that looked at a total of 17 studies with 634 patients using oral minoxidil as a primary treatment for hair loss (mostly AGA but including CIA) found that it was an effective alternative treatment for patients who had trouble using the topical minoxidil solution [46].

However, because vasodilation leads to a greater permanence of anticancer drug around the HF, minoxidil should not be used during chemotherapy but should be administered after chemotherapy discontinuation to achieve a greater regrowth rate [9]. To determine the optimal time to administer topical minoxidil, the pharmacokinetics of the chemotherapeutic drug needs to be considered, and a topical hydrocortisone should also be administered with minoxidil to act as an anti-inflammatory agent and support hair growth [9,44,47]. Interestingly, in murine studies, minoxidil not only significantly improved the hair quality (both length and growth) after paclitaxel treatment, but also suppressed neuroinflammation, as well as showing a synergistic anti-tumor effect with paclitaxel in tumor xenograft models of both cervical and breast cancer [49]. Therefore, minoxidil is an excellent option for treating CIA, as well as a potential candidate to prevent chemotherapy-induced peripheral neuropathy. There is an ongoing clinical trial (NCT03831334) evaluating the effect of low-dose oral minoxidil as treatment for pCIA (Table 2).

### 3.2. Bimatoprost/Prostaglandin F2α Analog

Bimatoprost is a synthetic analog of prostaglandin F2α. It increases the outflow of aqueous fluid from the eye and lowers intraocular pressure, and it has been used to treat conditions with high pressure inside the eye, including glaucoma [50,51]. Because the association of acquired trichomegaly of the eyelashes and hypertrichosis was discovered with bimatoprost use [52,53], bimatoprost (0.03%) has been used to treat eyelash hypotrichosis and eyelash, eyebrow, and scalp alopecia [54,55], as well as for aesthetics purposes. Several controlled clinical studies have shown the efficacy of bimatoprost gel for chemotherapy-induced ciliary hypotrichosis, reporting a faster regrowth and an increased density of treated lashes [55]. For example, in a multicenter, randomized, double-blinded, parallel-group study in Japanese female chemotherapy patients, bimatoprost-treated subjects showed significantly greater increases in eyelash length, thickness, and darkness at 4 months compared to vehicle-treated subjects (*p* ≤ 0.04, *N* = 36) [56]. In another multicenter, randomized, double-blinded, parallel-group study in predominantly white female chemotherapy patients, improved global eyelash assessment was made in 37.5% of subjects receiving bimatoprost versus 18.2% of subjects receiving vehicle (*p* = 0.04) at month 4 and 46.9% versus 18.2% (*p* < 0.01) at month 6 [57]. Adverse effects include conjunctival hyperemia, punctate keratitis, and eye pruritus, and were largely localized to the treatment area, mild in severity, reversible upon treatment cessation, and predictable based on the known pharmacology of bimatoprost [57]. These studies and others provide statistically significant evidence that bimatoprost 0.03% application once daily to the upper eyelids promotes eyelash regrowth after CIA in multiple racial groups.

Additionally, latanoprost, another prostaglandin F2α analog used to treat increased pressure inside the eye, was also found to stimulate eyelash growth and pigmentation [58]. It was further shown to also stimulate hair growth in pattern baldness [59,60], though its ability to stimulate hair regrowth after CIA remains to be investigated.

### 3.3. Photobiomodulation Therapy (PBMT)

Photobiomodulation therapy (PBMT), or low-level laser therapy, is largely used in medicine and dentistry due to its ability to accelerate healing by increasing cell viability [61,62]. Recent studies have illustrated that PBMT stimulates mitochondrial cellular respiration via increasing ATP synthesis by photodissociation of the inhibitory nitric oxide from the heme and copper center of cytochrome C oxidase [61,62,63]. While PBMT has been more widely used for wound healing and reduction of pain and inflammation in musculoskeletal disorders, it has gained momentum in the 21st century as a potent treatment for hair loss by inducing HFs to exit telogen and enter an active anagen phase [61,62,64]. Because of its excellent safety profile, non-invasive nature, relatively low costs, availability as home devices, and ease to us, PBMT is a popular option for patients and is used with high compliance rates [64]. PBMT has been shown to be effective in treating male and female pattern hair loss and alopecia areata [61,62,64,65], but its effectiveness on CIA has not been extensively explored. It is hypothesized that PBMT for patients with CIA may inhibit apoptosis in the HFs by upregulating anti-apoptotic proteins such as those in the mitochondria [61,66]. While PBMT using red lights (630–655 nm) has shown successful results in promoting hair density and hair growth in patients as well as in animal models, recent studies have found positive effects of blue lights (453 nm) on ex vivo hair growth [66,67,68,69,70]. This could be due to the presence of another group of chromophores, called opsins, within the HFs. Opsins are responsive to blue-light signals, reduce apoptosis, and prolong anagen; however, future studies are needed to study the effect of blue light in vivo [61,66]. Thus, while PBMT has been shown to be successful in treating CIA in rat models [71] and other types of alopecia in human studies, further investigation is needed concerning human CIA. Results from three current clinical trials will help determine the safety and efficacy of PBMT in promoting hair regrowth after CIA (Table 2).

### 3.4. Platelet-Rich Plasma (PRP)

Platelet-rich plasma (PRP), also called autologous platelet concentrates or APCs, is a concentrate of platelet-rich plasma proteins derived from the whole blood, prepared by removing red blood cells and a portion of plasma by centrifugation, sometimes followed by sonication [72]. PRP is “activated” when growth factors and cytokines are released. Whereas host dermal collagen and endogenous thrombin are able to activate PRP, calcium gluconate, calcium chloride, or exogenous thrombin can be added before administration (“activated PRP” or AA-PRP) [73]. Activation helps to release many growth factors, including platelet-derived growth factor (PDGF) type a and b, transforming growth factor (TGF) type α and β, vascular endothelial growth factor (VEGF), epidermal growth factor (EGF), fibroblast growth factor (FGF), connective tissue growth factor (CTGF), and insulin-like growth factor-1 (IGF-1) [73]. Additionally, platelets can also release numerous anti-inflammatory cytokines such as interleukin-1 receptor antagonist (IL-1ra), soluble tumor necrosis factor (TNF) receptor (sTNF-R) I, interleukin (IL)-4, IL-10, IL-13, and interferon γ [73]. PRP has been used as an autologous injection for various medical conditions to promote healing and tissue regeneration/rejuvenation [73,74,75,76,77,78]. Additionally, PRP has also been used widely in alopecia treatment due to its ability to induce cell proliferation, supporting HF growth, and extending anagen [72,79,80,81,82]. Whereas PRP treatment has the advantages of minimal invasiveness, low cost, and being autologous, its effectiveness has not been vigorously validated, partly due to the autologous nature and partly due to the variations in preparation protocols [83]. A study in rats investigated the effects of clinically relevant concentration and preparation of PRP on preventing CIA by cytosine arabinoside (Ara-C), etoposide (VP-16), and Cytoxan (cyclophosphamide) chemotherapeutic agents, but did not show any protection against CIA or promotion of hair regrowth afterwards [84]. Therefore, the effectiveness of PRP needs to be vigorously validated before its use can be expanded in the clinic. Additionally, PRP is relatively contraindicated in patients with hematologic malignancies. A pilot study on the effectiveness of PRP for the treatment of endocrine therapy-induced alopecia and pCIA in breast cancer patients (NCT04459650) is expected to complete shortly.

### 3.5. Spironolactone

Spironolactone is a synthetic aldosterone receptor antagonist that has been used off-label for various dermatological conditions [85,86,87]. It inhibits circulating testosterone by occupying the androgen receptor and thereby displacing androgenic metabolites such as 5 alpha-dihydrotestosterone, thus limiting the nuclear translocation and subsequent transcriptional activation by androgen receptor. While its antiandrogenic properties prevent spironolactone from being used by males, it is suitable to treat conditions in females in which excess androgen production leads to unwanted and psychologically distressing manifestations, including acne, hidradenitis suppurativa, hirsutism, and female pattern hair loss [85,88,89,90,91,92]. A recent study also explored using spironolactone to treat pCIA [11]. In a group of 54 patients, most of whom had breast cancer and developed pCIA after taxane-based chemotherapy, 36 (67%) showed a moderate to significant improvement after treatment with oral spironolactone combined with topical minoxidil or topical minoxidil alone [11]. While this study did not assess the effects of spironolactone alone on pCIA, a separate study showed that spironolactone alone was effective in promoting hair growth and inhibiting alopecia progression, at a dose of 200 mg/day [89]. There had been concerns about spironolactone regarding the hypothetical risk of hormonal stimulation of endocrine receptor-positive tumors [93]; however, recent studies suggest that spironolactone might actually be linked with reduced cancer incidents [94,95,96,97,98,99]. Nonetheless, more extensive research is needed to confirm the effectiveness of spironolactone in treating CIA and assess potential adverse effects.

### 3.6. Other Agents

Besides the approaches discussed above, a variety of other topical and systemic agents have also been tested to prevent or treat CIA. Cyclosporine, an immunosuppressive calcineurin inhibitor that is known for inducing hypertrichosis, has been shown to not only induce active hair growth but also inhibits regression of HF in experimental models [100]. Murine studies have shown that with the use of etoposide, cytarabine, cyclophosphamide, and doxorubicin, both oral and topical cyclosporine can induce regrowth and slow hair loss, as well as prevent already damaged HFs from progressing into the terminal telogen phase [100]. Cyclosporine was also shown to prolong anagen in organ-cultured human HFs, suppressing the Wnt inhibitor SFRP1 in the dermal papilla [101]. In addition, topical calcineurin inhibitors such as tacrolimus and pimecrolimus have shown limited effectiveness in the treatment of alopecia areata [102] and frontal fibrosing alopecia [103,104]. Animal studies also showed that topical tacrolimus induces anagen and protects from CIA [105], although its effectiveness in patients remains to be assessed.

Furthermore, antioxidants are postulated to be effective against chemotherapy-induced toxicity due to their ability to eliminate free radicals and other reactive oxygen species created by chemotherapy [106,107]. In a rat model of CIA, topical application of edaravone ointment of 3% or higher led to decreased hair loss after cyclophosphamide chemotherapy [106]. Furthermore, when a novel antioxidant M30 [5-(N-methyl-N-propargylaminomethyl)-8-hydroxyquinoline]—a multi-target iron chelator—was given to mice injected with cyclophosphamide, elevated hair growth, as well as inhibited growth of cyclophosphamide-induced abnormal hair, were observed [108]. Moreover, 0.5–1% sodium zinc dihydrolipoylhistidinate (DHLHZn) applied topically to rat skin reduced the inflammatory cell infiltration around the HFs induced by cytosine arabinoside [109]. A more recent study in mouse models injected with cyclophosphamide showed that DHLHZn treatment preserved the level of insulin-like growth factor-1, an anagen promoter, and improved hair bulb diameter, leading to decreased alopecia [110].

### 3.7. Complementary and Alternative Treatments

Due to the varied efficacy and application regimen of current treatments for alopecia, many patients have turned to complementary and alternative medicine (CAM) for safe and natural alternatives. Many natural products have been claimed to increase hair density, with most studies conducted for androgenetic alopecia and alopecia areata. Natural products that have been suggested to be effective for alopecia treatment include amino acids (N-acetyl-L-cysteine, L-cystine, lysine), caffeine, capsaicin, curcumin, garlic gel, marine proteins (including extracellular matrix components from sharks and mollusks), melatonin, onion juice, procyanidin flavonoids, pumpkin seed oil, rosemary oil, saw palmetto, vitamin B6, vitamin B7 (biotin), vitamin D, vitamin E derivatives, zinc, and oral glucosides of peony with compound glycyrrhizin [111,112]. L-cystine combined with vitamin B6 have also been shown to effectively prevent doxorubicin-induced alopecia in mice [113]. In addition, hypnosis, essential oil aromatherapy, acupuncture, massage, electromagnetic stimulation, mindfulness psychotherapy, and homeopathy have also been proposed to improve psychologic and quality of life outcomes for alopecia patients [22,111,112]. Some of the studies on CAM effects lacked strong evidence, and more rigorous studies are needed to prove efficacy. Encouragingly, a topical lotion, CG428, made from botanical products was recently shown in a randomized double-blind controlled trial (NCT02605629) (Table 2) to promote hair density and thickness in breast cancer survivors with pCIA [114]. CG428 is a botanical product containing a patented blend of four botanical ingredients—citrus, cocoa, guarana, and onion [114]. After 6 months of application (twice daily), participants in the CG428 group showed a higher average hair density increase than the placebo group, and were more likely to report improvement of overall volume, hair density, and thickness, although these differences were not statistically significant [114]. It was speculated that CG428 may reduce apoptosis within the HFs through increasing the expression of anti-apoptotic Bcl-2, thereby prolonging the anagen phase and increasing the anagen:telogen HF ratio, allowing the new anagen hair to grow longer and to a thicker diameter [114]. Two other clinical trials on CG428 (NCT02919735 and NCT02986412) were recently completed, but the results are yet to be published (Table 2).

## 4. Supplementary Management

With hair loss often being seen as a sign of poor health or cancer treatment and its high incidence rate after chemotherapy [4], the anxiety and anticipation of chemotherapy causes tremendous psychological stress on patients, even influencing up to 14% of patients to consider rejecting chemotherapy and 8% of patients to completely reject chemotherapy [4,18,115]. Qualitative studies have also shown that many women experiencing breast cancer view CIA as being more distressing than losing a breast [116,117,118]. Thus, it is important to explore supplementary care and management through multidisciplinary approaches. Patients experiencing negative psychological concerns should be advised to seek psychological support through therapy or patient associations or support groups [21,119]. For aesthetic purposes, which play a large role in a patient’s QoL, other forms of supplementary management include the use of turbans, head scarfs, bandanas, and wigs [21,120]. Other camouflage techniques include cutting one’s hair shorter early on, keratin powder, and dermopigmentation [21,116,121,122]. Keratin powder uses natural keratin that attaches to a patients remaining hair or hair that is growing back via static electricity, making the hair appear fuller and denser temporarily [11,21]. Dermopigmentation is a pigment injection technique that deposits bioresorbable pigments into the epidermis and superficial dermis [21,121,122]. This is a temporary but long-lasting method that gives the illusion of increased hair density for 2 to 5 years and can be particularly useful to camouflage alopecia of the scalp or eyebrow [21]. When looking into the future, with the expected large increase in cancer survivors, it is important to find other methods of palliative care that can help increase a patient’s QoL while dealing with and recovering from CIA, taking into consideration the psychological impact of hair loss on these patients.

## 5. Conclusions

**Figure 1:**
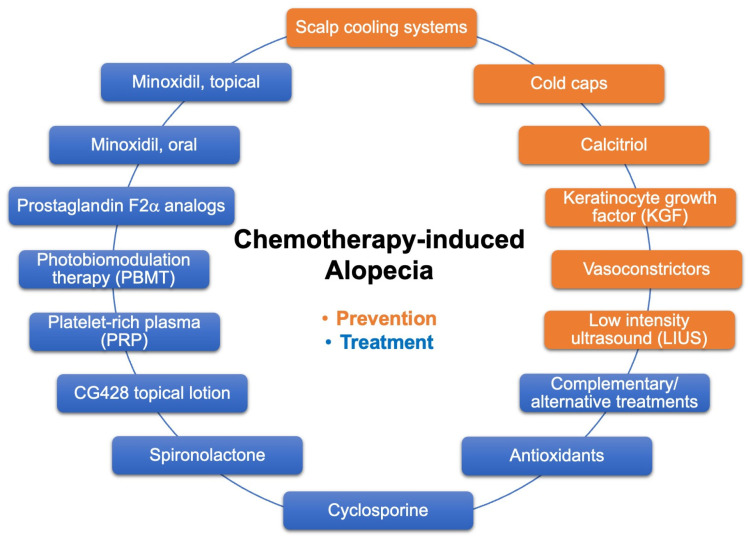
Current approaches to prevent and treat CIA.

The current approaches to prevent and treat CIA are summarized in Figure 1. Some of the devices/agents are being studied in clinical trials (Table 2), and positive results may bring new options for cancer patients facing chemotherapy. Presently, scalp cooling is the most effective strategy to **prevent CIA** for patients with solid tumors undergoing certain chemotherapy regimen. Using FDA-cleared scalp cooling systems, scalp cooling has high patient compliance, and can reach a mean success rate of 50–70%. Self-administered cold caps, not cleared by the FDA, are also available from several manufacturers. However, scalp cooling is not suitable for patients with hematological tumors or certain peripheral neuropathies, or for patients prone to cold injury, and costs and access remain an issue. Even in countries with universal healthcare systems, the extra time needed for the patient to stay in the chemotherapy room is a drawback. For those patients who cannot use scalp cooling, application of topical agents such as calcitriol, KGF, and vasoconstrictors may offer good alternatives. Topical agents also have the advantage of being applied as often as daily instead of only during infusion time for scalp cooling.

For **CIA treatment**, minoxidil is by far the most effective agent to promote hair regrowth. Topical minoxidil has been found to shorten the alopecic period and promote hair regrowth after chemotherapy, while low-dose oral minoxidil has been shown to be an effective alternative for those who have difficulty using topical minoxidil. It is important to note that minoxidil should not be used during chemotherapy due to its vessel dilating effects but should be used after chemotherapy cessation. For eyelash regrowth, bimatoprost (0.03%) has shown efficacy in several studies, reporting increased eyelash length, thickness, and darkness in treated subjects compared to control. Additionally, several other treatment methods have been shown to promote hair regrowth in alopecia areata and androgenetic alopecia, and in animal studies against CIA. However, their effectiveness for treating CIA patients requires further investigation. Among them, PBMT stimulates mitochondrial cellular respiration and has been used for hair loss treatment by inducing HFs to enter an active anagen phase. PRP contains an abundance of growth factors that can induce cell proliferation and support HF growth, and therefore promote healing and tissue regeneration. CG428, spironolactone, cyclosporine, and antioxidants could potentially offer promising results in the treatment of CIA in the future. A combination of physical and chemical interventions may help to compensate for the shortcomings of either therapy alone. Additionally, given the importance of scalp and facial hair as a sign of good health, beauty, and youth, CIA has a significant negative impact on patients’ self-esteem, body image, sexuality, and overall QoL. Therefore, it is important to advise patients to seek psychological support and provide them with supplementary management methods such as wearing wigs, turbans, head scarfs, and using camouflage techniques.

Globally, new cancer cases are projected to reach 28.4 million in 2040 [123]. With prolonged life expectancy, earlier and more accurate diagnosis and more targeted treatment, the number of cancer survivors is expected to increase tremendously. In the U.S. alone, the number of cancer survivors is projected to grow from 18.1 million in 2022 to 26.0 million by 2040 [124]. Oncology teams who recognize the effects of CIA and work with dermatologists would better address CIA and provide better overall care to patients, and likely improve cancer treatment compliance and outcome.

## Figures and Tables

**Table 1:** Comparison of the different scalp cooling devices used by patients with solid tumors undergoing chemotherapy.

Device	Date of FDA Clearance	Device Description	Temperature Control by Computer	Advantages	Adverse Effects	Operator	Manufacturer	Costs
DigniCap C3 Scalp Cooling System	2015 (for breast cancer) *	mobile cooling unit that uses circulating cold coolant	X	• The wrap is fitted at the beginning of treatment and remains on until completion • Starting from room temperature: comfortable	feeling of coldness, headache, scalp pain and/or light-headedness, dizziness	health-care providers administered	Dignitana	~USD 1500–2000
DigniCap Delta Scalp Cooling System	06/27/19						Digitana	~USD 1500–2000
Paxman Scalp Cooling System	04/19/17, later expanded to solid tumors						Paxman Coolers Limited	capped at USD 2200
Amma Cooler Heads cold cap	12/08/21						Cooler Heads	~USD 2000 rental costs
Penguin Cold Caps	not yet **	3-cap system: Crylon Gel in the caps	no	• Portable • Caps chill fast		self-administered	Penguin Cold Caps	USD 419/month rental costs
Chemo Cold Caps	not yet	6-cap system: caps filled with coolant gel					Arctic Cold Caps LLC	USD 379/month rental costs
Arctic Cold Caps	not yet	8-cap system: caps filled with glycerin-based hydr0-gel that refreezes in 2 h					Arctic Cold Caps LLC	USD 379/month rental costs

* 07/03/17 FDA-cleared for expanded use in cancer patients with solid tumors. ** All materials used in the manufacture of Penguin Cold Caps are approved in Canada and by the FDA in the USA.

**Table 2:** Current clinical trials for the prevention and treatment of chemotherapy-induced alopecia (clinicaltrials.gov).

Category	NCT Number	Interventions	Completed	Recruiting	Not Yet Recruiting	Device	RCT, Parallel Assignment	Single Group Assignment	Open Label	Gender	Target Sample Size/Enrollment	Prevention	Treatment
Scalp cooling	NCT01831024	Dignicap System	*			X	Non-R CT		X	Female	110	X	
	NCT03712696	Device: DIGNICAP™	X			X		X	X	Female	139	X	
	NCT04630080	Scalp cooling		X		X		X	X	Female	100	X	
	NCT05213936	Scalp cooling with hairstyle using conditioner and water emulsion		X		X	Non-R CT		Single blind (Investigator)	All	30	X	
	NCT03248193	Concomitant limb cryocompression and scalp cooling	*			X	Non-R CT		X	All	50	X	
	NCT04986579	Paxman Scalp Cooling System		X		X	Non-R CT		X	All	120	X	
	NCT01008774	Paxman Cooling Machine; Cold Caps	X			X	Non-R CT		X	All	239	X	
	NCT04180579	PAXMAN Scalp Cooler			**	X		X	X	Female	34	X	
	NCT04764357	Paxman Scalp Cooling System		X		X		X	X	All	40	X	
	NCT04117815	Paxman Scalp Cooling System	X			X	***			Female	128	X	
	NCT04626895	Paxman Scalp Cooling Device	X			X	X		X	Female	15	X	
	NCT05533320	Paxman Scalp Cooling System			X	X		X	X	Female	30	X	
	NCT04678544	Paxman Scalp Cooling System 2	X			X	X		Single blind (Outcomes Assessor)	Female	170	X	
	NCT04168242	Scalp cooling Paxman Orbis II system			**	X	X		X	Female	80	X	
	NCT01986140	PAXMAN Orbis Scalp Cooler Treatment with Orbis scalp cooling cap			**	X	X		Single blind (Care Provider)	Female	236	X	
	NCT03289364	Penguin Cold Caps	*			X		X	X	All	9		
	NCT05484973	AMMA Portable Scalp Cooling System			X	X		X	X	Female	125	X	
	NCT05365243	AMMA Portable Scalp Cooling System			X	X		X	X	Female	12	X	
	NCT03711877	Scalp cooling system; chemical cold cap		X		X	X		X	Female	256	X	
PBMT	NCT05177289	Theradome^®^ LH80 pro combined with scalp cooling		X		X	X		Single blind (Outcomes Assessor)	Female	72	X	X
	NCT04036994	Photobiomodulation therapy		X		X	X		Single blind (Participant)	Female	30		X
	NCT05397457	Low-level light therapy		X		X	X		X	Female	88		X
Topical	NCT02919735	Topical CG 428 herbal medicinal solution	X				X		Double blind (Participant, Investigator)	Female	40	X	X
	NCT02986412	Topical CG 428 herbal lotion	X					X	X	Female	19		X
	NCT02605629	Topical CG 428 herbal lotion	X				X		Double blind (Participant, Investigator)	Female	32		X
	NCT04554732	Topical Keratinocyte growth factor	X					X	X	Female	28	X	
	NCT01588522	Topical compound 31, 543 Calcitriol	X					X	X	All	30	X	
Oral	NCT03831334	Drug: oral minoxidil		X				X	X	All	25		X
Injection	NCT04459650	Platelet Rich Plasma			**			X	X	All	30		X
-	NCT02530177	Clinical Assessment of alopecia and pCIA, skin aging and nail changes: Observational			**		****			Female	546	-	-

*: Unknown status; **: Active, not recruiting; ***: Observational Model: Cohort; ****: Observational Model: Case-Control. RCT: Randomized controlled trial; Non-R CT: Non-randomized controlled trial. Note: NCT00999557 to evaluate the efficacy of topical Bimatoprost in promoting eyebrow and eyelashes has been withdrawn. NCT02797223, an interview-based quality of life study supposed to be completed by December 2016, was excluded.
